# Implementing breathlessness self-management in low- and middle-income countries: co-design of breathlessness self-management resources for use in India

**DOI:** 10.1038/s41533-025-00458-2

**Published:** 2025-11-24

**Authors:** Joseph Clark, Naveen Salins, Mark Pearson, Mithili Sherigar, Seema Rao, Siân Williams, Anna Spathis, Rajani Bhat, David C. Currow, Srinagesh Simha, Miriam J. Johnson

**Affiliations:** 1 Wolfson Palliative Care Research Centre, University of Hull, Hull, UK; 2 Department of Palliative Medicine and Supportive Care, Kasturba Medical College Manipal, Manipal Academy of Higher Education, Manipal, India; 3 Karunashraya Hospice, Bangalore Hospice Trust, Bengaluru, India; 4 Manipal Hospice and Respite Center, Manipal Academy of Higher Education, Manipal, India; 5 International Primary Care Respiratory Group, London, UK; 6 University of Cambridge, Cambridge, UK; 7 SPARSH Hospital, Bengaluru, India; 8 Flinders Ageing Alliance, Flinders University, Bedford Park, South Australia Australia

**Keywords:** Health care, Health humanities, Medical humanities

## Abstract

Breathlessness is prevalent in societies worldwide, with widespread health and socioeconomic impacts. Breathlessness self-management interventions developed in high-income countries (HICs) are promising but require contextual adaptation for low- and middle-income countries (LMICs) like India, where cultural beliefs, language, and delivery systems differ. We co-designed breathlessness self-management resources for use in India using a programme theory approach and Community-Based Participatory Research methods. We convened three stakeholder groups (Doctors (n = 9), Nurses and allied health (n = 6) and lived experiences (n = 9)) and added a fourth group (community health workers (n = 6)) based on emerging findings. We re-analysed 104 academic and lay sources identified iteratively and systematically by the Breathe-India project and presented evidence to stakeholder groups for discussion and feedback. Three rounds of online/face-to-face stakeholder workshops. Stakeholders reviewed evidence, developed shared definitions, and iteratively co-designed intervention components. Stakeholder engagement and evidence synthesis led to identification of seven key domains informing the intervention: (1) Identifying breathlessness— teach the difference between acute and persistent breathlessness (and acute-on persistent breathlessness); (2) Developing shared language—emphasising lived experience of breathlessness in simple, translatable language; (3) Addressing fear—teaching accessible methods (e.g. facial cooling) for regaining control that build confidence; (4) Building resilience—reframing activity as safe and beneficial; (5) Daily coping strategies—aligning with local beliefs and behaviours, e.g. inclusion of nutritional ‘dos and don’ts’; (6) Delivery through community infrastructure—teaching Accredited Social Health Activists (ASHAs) how to identify breathlessness in communities and challenge unhelpful beliefs—at the point of care. Outputs included training curricula, educational resources, and public-facing materials co-developed with ASHA trainers and stakeholders. We co-designed India’s first multicomponent, community-deliverable breathlessness self-management intervention using participatory methods and theory-driven processes. Implementation-effectiveness hybrid evaluation is needed to test feasibility, acceptability, and impact on patients and families.

**Chronic breathlessness** is caused by many communicable and noncommunicable diseases (NCDs)^1^, and often persists despite treatment of causal disease(s)^2^. It affects over 90% of people with Chronic Obstructive Pulmonary Disease (COPD)^3^, advanced heart failure^4^, and advanced lung cancer^5^. Most diseases causing chronic breathlessness are prevalent and increasing in low- and middle-income countries (LMICs)^6^.

Evidence from high-income countries (HICs) shows that breathlessness can be perpetuated by physical deconditioning, anxiety and breathing pattern disorders. It profoundly impacts day-to-day function, emotional wellbeing and family life. Over 50% of people with breathlessness also have anxiety (cause or effect)^7^. Breathlessness is associated with premature mortality, lower health-related quality-of-life (HRQoL) and higher disability scores^8^. Those affected contribute less to the workforce^9^ and have lower household income risking – or entrenching – poverty^10^.

In LMICs, similar challenges are present and may be far more widespread and damaging in the context of higher poverty and environmental factors (e.g. air pollution, heat)^11^. A cross-sectional study conducted in India identified that 44% of the general population report breathlessness limiting exertion (modified Medical Research Council breathlessness scale (MMRC) ≥1)^12^. This is a markedly higher prevalence than we see in HICs^13,14^. Quantitative evidence indicates that people reporting breathlessness have higher disability scores and lower quality of life^9^. They are less likely to participate in the workforce or struggle to do their work, with direct and indirect economic consequences for the person and their families^15^. People in low paid roles, especially in rural areas are more disadvantaged because of the physical demand of their work, or exposure to risk factors (e.g. autorickshaw drivers)^15^. However, despite the scale and severity of the problem of breathlessness, few symptom-focussed services are available and most need goes unaddressed^16^.

In HICs, there is increasing evidence for the value of a range of breathlessness self-management interventions (e.g. handheld fan^17,18^ breathing exercises^19,20^) Such interventions are relevant to LMICS because they are low-cost, feasible to implement and congruent with traditional address a known problem, are effective and are low cost at the point of delivery, meaning they have high potential to be implemented equitably and sustainably^21^. However, self-management interventions have not been tested for acceptability, implementability or effectiveness in LMICs.

There is increasing evidence that health interventions developed in high-income settings may not be helpful in LMICs if not adapted to context. For example, a project conducted in Bangladesh identified that effectiveness evidence for pulmonary rehabilitation is highly applicable between HICs and LMICs, but that effectiveness can be lost during implementation^22^. Unhelpful beliefs and behaviours are those which may worsen outcomes, often inadvertently and must be addressed during implementation. International collaborations and participatory methods are important mechanisms regarding how co-design can translate available evidence into developing acceptable, feasible, and adaptable health solutions in different settings. For example, the NIHR Global Health Research Group on Respiratory Rehabilitation worked with communities in Kyrgyzstan and incorporated community practices (rhythmic movement) into delivery of pulmonary rehabilitation to enhance acceptability^23^. A further project conducted in Sri Lanka, incorporated nutrition, singing and dance into delivery of pulmonary rehabilitation to enhance acceptability^24^.

To improve breathlessness self-management in India, we conducted the Breathe-India project; a realist review and co-design project which aimed to: 1) develop intervention and implementation programme theories; and 2) co-design a study intervention with stakeholders. In this paper, we report our co-design work. Our co-design objectives were to:

- To develop shared understanding and a contextually relevant language for breathlessness among healthcare professionals, community health workers, people with lived experience, and families in India.
- To identify and adapt evidence-based breathlessness self-management strategies from high-income countries for feasibility and acceptability in India.
- To co-produce prototype breathlessness self-management resources and training curricula tailored for community delivery in India, particularly through Accredited Social Health Activists (ASHAs).

Four components of co-production are delineated: commissioning, designing, delivering and assessing^25^. We conclude with our future plans for implementation-effectiveness research, which will address co-delivery and outcome assessment.

## Methods and study design

### Evidence generation and theory development

Our process of evidence generation is presented in full elsewhere^23^. In brief, we drew on findings from our realist review and conducted further analysis of 104 included published sources and stakeholder involvement to identify helpful breathlessness interventions and programme theory^26^ regarding feasibility of implementation in community settings in India. We used systematic and iterative searches to identify literature relevant to breathlessness management in Asia, supplemented by key papers recommended by our Project Management Group and International Steering Group. Sources were read in detail and aspects of theory were extracted. Included interventional studies were assessed with regards to the intervention’s effectiveness, components, cost and place and mode of delivery. We drew heavily on the International Primary Care Respiratory Group’s Desktop Helper, a suite of resources recommended for implementation in primary care worldwide^27^. We excluded breathlessness interventions that required specialist delivery (e.g. by a medical professional).

### Intervention co-design and stakeholder involvement

Our co-design approach was grounded in the principles of Community-Based Participatory Research (CBPR), emphasizing equitable collaboration with stakeholders from clinical, lived experience and community health domains^28^. Stakeholders were engaged as partners throughout all stages of the project to ensure that programme theories and interventions were contextually appropriate, culturally relevant, and appropriate for use in the context of existing community health infrastructure. Our discussions were informed by four domains of co-production; commissioning (needs assessment and defining the population), design (intervention development), delivery (workforce development) and assessing (evaluation)^26^. In this paper, we focus primarily on design and delivery and outline our plans for evalution.

We convened three stakeholder groups in India (doctors (n = 9), nurses and allied health professionals (n = 6), people with lived experiences of breathlessness (n = 9) [people living with, or family members caring for someone with, breathlessness]) and an International Steering Group to co-develop programme theory and prototype breathlessness resources. Stakeholder group composition is presented in Supplementary File 1. Informed by our emerging findings about the important role of Accredited Social Health Activists (ASHA) workers in supporting communities, we added a fourth Indian stakeholder group of ASHA workers (n = 6). Stakeholders were invited to join the project as partners (not research participants) to provide their views on evidence generated by our research team. Stakeholders were offered a small monetary thank you for their contributions in workshops lasting between 60–90 min.

### Stakeholder workshops

We conducted three rounds of stakeholder workshops, with additional stakeholder informal feedback throughout (e.g. 1-1 conversations, reading resources and providing comments). Stakeholder workshops were conducted online, in the English language, led by three different members of the project management group based in India (NS, SR, RB). A researcher (JC) and study coordinator (MN) were present and took notes. Group meetings with doctors, nurses, allied health professionals, and people with lived experience were convened separately to minimise risk of power differentials inhibiting participation. Workshops with ASHA workers were conducted in the Kannada language, by NS and notes were taken by MS. Steering Group meetings followed each round of Stakeholder workshops.

Each round of workshops followed the same structure. Evidence was reviewed by the research team and theories generated. Theories and intervention components were presented to stakeholders for their views with aims for the workshop and questions to promote discussion. An example is presented in Table 1.

**Table 1:** Example workshop materials.

Aim	Definition	Questions
To understand what we mean by ‘persistent breathlessness’?	Persistent breathlessness is: Breathlessness that persists despite treatment of causal disease(s) Persistent breathlessness causes disability and a vicious cycle of avoiding physical activity, deconditioning, anxiety and increasing breathlessness	Is this description of ‘persistent breathlessness’ meaningful for use in the context of India? Are there any issues surrounding the language for breathlessness which we should be aware of?

**Fig. 1:**
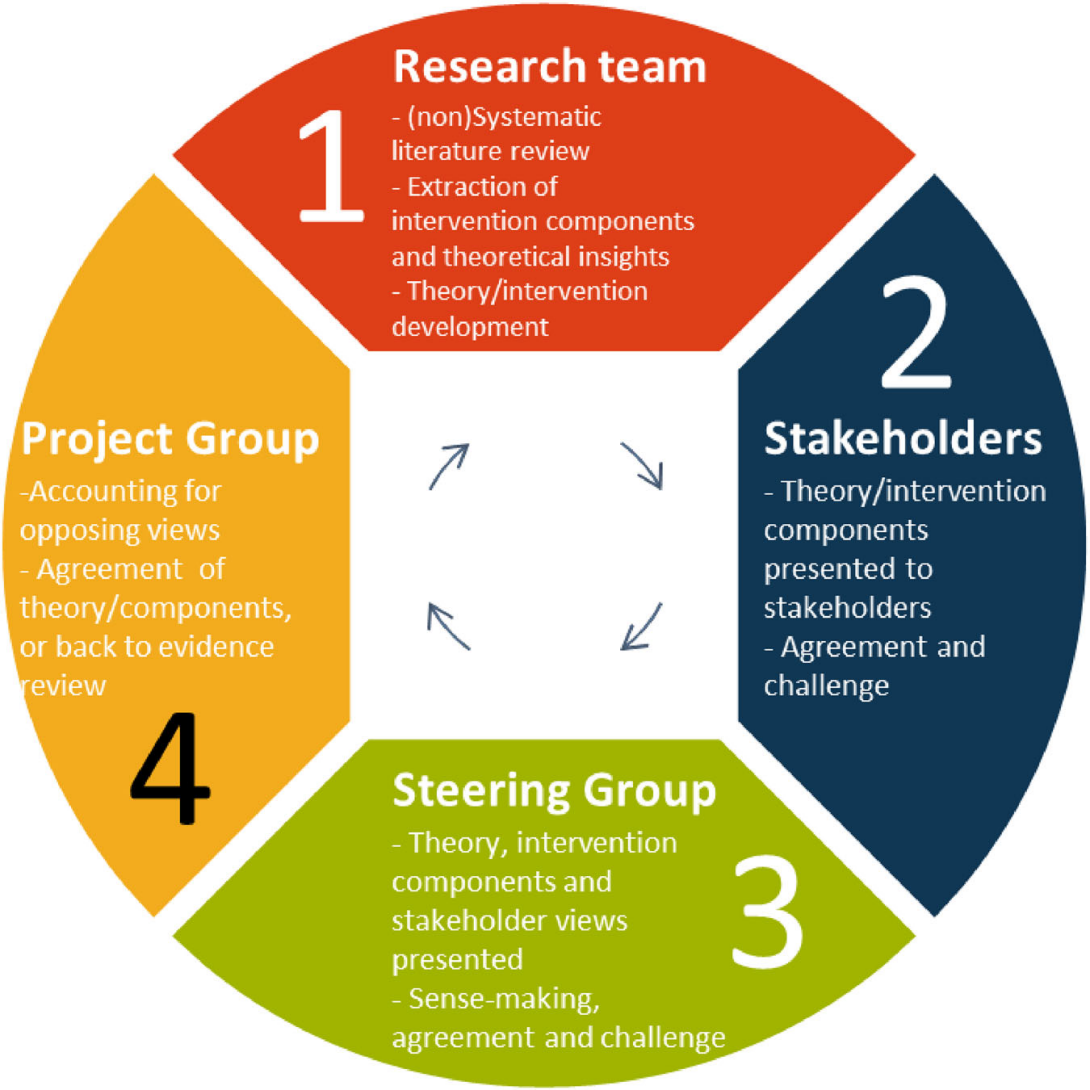
Study decision-making process.

Stakeholders were asked to draw upon their experiences to offer their views about whether theories or intervention components would be acceptable to health workers, people with breathlessness and their families. Views were presented to the Steering Group, for their support in decision-making and addressing inconsistencies. Agreed, contested and new evidence was then presented back to stakeholders for their views. Our decision-making process is summarized in Fig. 1.

Our iterative approach informed the decision to convene a further Stakeholder Group of ASHA workers to explore the appropriateness of programme theories and review the intervention in-development. Once intervention components were agreed, JC organized the components into three breathlessness resources. The contents, language used and supporting images (created by a local illustrator, aligned with the intervention components) were then reviewed by selected members of each Stakeholder Group, the Steering Group and finally agreed by the research team. The final breathlessness resources we developed were consistent with our programme theories, developed during our realist review.

Our co-design work drew on evidence used to co-develop programme theories relating to; context, intervention and implementation. Full programme theories are presented in Clark et al.^22^, a summary is provided in [Box 1].

Box 1 Breathe-India programme theories **Context**

Breathlessness is common and often misunderstood as aging, asthma, or hereditary. It is stigmatized and shaped by cultural beliefs. People avoid activity due to fear, and seek simple, trusted guidance, though both families and health workers may hold unhelpful beliefs.

**Intervention for breathlessness**

Recognizing breathlessness as a real, treatable issue can reduce fear. Simple self-management tools that promote safety and behaviour change are effective across settings.

**Implementation strategies**

ASHAs, as trusted community health workers, are well-placed to deliver simple, evidence-based interventions that challenge stigma and support appropriate healthcare use.

## Results

An evidence review, combined with equitable collaboration among clinical experts and community members with lived experience, led to the development of six key domains, considerations, and recommended responses to be included in breathlessness education design and delivery. Summary findings are presented in Table 1 and domains are reported in full below.

**Table:** 

Domain	Consideration	Response
Identifying breathlessness	Breathlessness may have multiple, unaddressed causes. Symptom management shouldn’t discourage appropriate health-seeking.	Teach the public and health workers the difference between acute and persistent breathlessness (and acute-on persistent breathlessness). Encourage health-seeking for unknown causes.
A language for breathlessness	Breathlessness is interpreted differently across groups. Health workers may use medical terms that assume causes have been addressed. People with lived experience focus on sensation and functional and emotional impacts.	Use person-centred, and simple language which reflects the pervasive impacts of breathlessness and can be translatable across settings.
Addressing fear – recovering breathing control	Breathlessness is frightening. People avoid activities they believe will trigger it.	Teach accessible methods like facial cooling, breathing techniques, and positioning. These help regain control and build confidence when breathless.
Increasing resilience to breathlessness	Avoidance behaviours can worsen breathlessness. Breathlessness from exertion is often misunderstood as being harmful. Exercise is perceived as “sport.”	Reframe breathlessness as a healthy exertion response. Emphasise that too much rest can worsen symptoms. Strategies include “move more, sit less,” pacing, and walking aids. Use of handheld fans to aid recovery can reinforce that breathlessness is safe and manageable.
Living with breathlessness	Interventions must align with local beliefs, behaviours, and environments.	Offer practical advice for daily coping. Highlight the impact of unhelpful behaviours (e.g. smoking) and promote positive change (e.g. if the person smokes tobacco, offer Very Brief Advice^29^. Include culturally relevant nutritional advice to engage with local health beliefs and strengthen intervention credibility.
Mode of delivery	Breathlessness may not be reported unless actively asked about. Delivery must align with local health systems.	Teach ASHA workers how to identify hidden breathlessness cases through proactive family engagement. Address unhelpful beliefs—both among families and health workers—at the point of delivery.

### Identifying breathlessness

Our programme theories indicated that despite high prevalence and negative impacts of breathlessness, people do not seek healthcare for breathlessness. To support health workers in identifying breathlessness of any cause, we identified the validated screening question ‘Have you suffered with breathlessness for most days in the last month’?^30^ Treating family as the unit of care, we agreed with our Steering Group the question could be adapted to ‘has anyone in your household suffered breathlessness for most days in the last month?”

Breathlessness may be caused by multiple long-term conditions, some, all, or none of which may be medically treated (Doctors Group). Many people who report breathlessness in India do not know what causes their breathlessness, or attribute it to poverty-related factors (e.g. malnutrition). Because of this complexity, a breathlessness intervention should not assume optimal management of underlying cause(s) and should include promotion of seeking a diagnosis alongside symptom management, where cause(s) of breathlessness may be unknown – and potentially reversible.

Breathlessness of unknown cause raises a challenge for symptom-focussed interventions. All stakeholder groups and the research team agreed that defining underlying causes was paramount to addressing modifiable causes widespread in India (e.g. anaemia). Additionally, concern was expressed at a theoretical risk that breathlessness management could ‘mask’ underlying cause(s), unintentionally delaying attending a qualified health practitioner for a diagnosis. Our Nurse and Allied Health Group also noted that panic attacks commonly lead to acute hospital attendances but could safely be managed with self-management. Our Steering Group agreed that masking health problems through symptom management was a minor risk, but still a consideration.

It was agreed by the Doctor Group and Steering Committee that a symptom-focussed intervention should include ‘red flags.’ That is, to support health workers identify the difference between persistent breathlessness and acute breathing problems. It was further agreed that development of diagnostic pathways using breathlessness as a tracer condition could be fruitful, but that this was out of scope for the development of a symptom-focussed intervention.

### A new language for breathlessness

Ensuring that all stakeholder groups had a shared understanding of problematic breathlessness was a key challenge. All Stakeholder Groups highlighted how there is no common non-professional language for breathlessness in India. Dialectical, or regional terms for breathlessness were highlighted as being likely to vary significantly - even between short geographic distances and within communities. Our evidence was presented in English, and it was noted by the Doctors Group and Nurse and Allied Health Group, that people in South India were more likely to speak English, risking widening inequalities. Stakeholders recommended that any terms agreed by the study team should be simple, making it more likely that they can be directly translated to other Indian languages.

Our initial definition was ‘breathlessness that persists despite treatment of causal disease(s), causing anxiety, deconditioning and worsening breathlessness [Table 1]’. All stakeholder groups agreed that these mechanisms were present but found the assumption of optimal management and the language used problematic. Key objections to the definition presented, were that:

i. persistent breathlessness may not be in context of disease (or optimal management – which may not be available, or accessible even if the person was diagnosed with a chronic condition);
ii. it may be from other causes e.g. frailty; and
iii. anxiety was not the only emotional trigger for breathlessness and preferred the term ‘distress’ to capture experiences.

The Doctors Group and Nurse and Allied Health Group both noted overlapping terminology; ‘chronic breathlessness,’ ‘continuing breathlessness’ and ‘refractory breathlessness.’ Doctors felt that these terms did not adequately convey the meaning regarding duration of breathlessness as lay people often did not understand ‘chronic’ in terms of time, and noted that persistent breathlessness need not be continuous. Several Doctors associated ‘persistent breathlessness’ with oncology, and preferred the term ‘refractory breathlessness’. Others disagreed and stated that persistent breathlessness is more meaningful, and that ‘refractory’ may indicate therapeutic nihilism. People with Lived Experience reported that they did not use medical language to refer to their breathlessness, more commonly referring to the sensation of breathlessness. Examples given were ‘lack of oxygen,’ or ‘I can’t get enough air.’ Another stakeholder referred to ‘not able to take the full amount of air, like there is a blockage.’ Despite this, all were confident that they understood medical definitions if explained by a medical professional.

### Addressing Fear – methods for recovering breathing control

Breathlessness presents ongoing challenges for undertaking daily life. However, stakeholders in all groups all focussed first on breathlessness as a frightening experience – for the person, their families – and their health workers^31^. Our programme theories emphasise that people need to feel ‘safe’ before they may find interventions aimed at increasing their fitness to be acceptable.

#### Handheld fans

Use of a handheld fan is effective at improving breathlessness and reducing recovery time following exertion^17,32^. Our Nurse and Allied Health Group confirmed that they use fans in their clinics, predominantly for people with COPD. Fans were noted to be widely available, at low cost (although some concerns were noted regarding battery life for sustainability of the intervention). The Doctors Group noted that facial cooling, using a damp cloth achieves similar effects. However, concerns were raised regarding acceptability about cooling, in context of health beliefs in communities which associate cold temperatures with cough, sputum production and worsening of respiratory illness^33^.

Fans are widely use in India, but with the primary purpose of cooling down – not breath recovery. Cooling, or cold, has strong associations with poor health in India, through the lens of indigenous health beliefs which may influence acceptability. Concerns were also raised about whether people would accept fans as a health intervention, given pre-existing associations and expectations of medicine for health problems. However, fans were reported as being increasingly used in pulmonary rehabilitation, although availability remains scarce^34^. Palliative care clinicians also reported their use in the Doctors Group and people in the Lived Experience Group reported finding them helpful. Stakeholders in all groups endorsed a ‘give it a go’ approach. Fans/facial cooling reduces breathlessness recovery time^19^ and should be promoted as ‘part of modern medicine’ alongside acknowledgement of pre-existing associations.

#### Breathing exercises

Breathing exercises (e.g. rectangle breathing^25^, pursed-lip breathing^35^ are effective in improving breathing control. Other activities (e.g. singing^36^ also show benefits for breathing control. Breathing exercises have a long history of use in India, in particular *Pranayama* as an aspect of yoga although perceived association of Yoga interventions with a religion made it less acceptable for people from other religions to adopt^37^. However, we also discussed practices from other religions and communities (e.g. chanting, singing). Our Steering Group agreed that presentation of breathing exercises *without* cultural connotations, would promote delivery of essential information and leave individuals to draw their own associations (which may be enabling).

Stakeholders in all groups confirmed that breathlessness could be a frightening experience, invoking a panic response commonly leading to hyperinflation of the lungs as people strive to ‘get enough oxygen.’ Emphasis on teaching people to focus on the **out** breath may be counterintuitive to people who feel they need *more* air but is understandable when framed as ‘making space for new air.’

#### Body positioning

Positioning the body in a comfortable position is helpful for regaining breathing control^38^. Body positioning in optimal positions can increase respiratory muscle efficiency and decrease energy expenditure to help breathlessness. No barriers were identified to this intervention component. Providing images portraying examples of positioning approaches that could be used even when out in the community was thought to be helpful.

### Increasing resilience to breathlessness

Addressing fear as a key driver of avoidance behaviour which decreases resilience to breathlessness creates opportunities for helpful breathlessness behaviours. We identified a range of strategies which can increase resilience to breathlessness. Notably, pulmonary rehabilitation was noted as an intervention which can help breathlessness^22^. However, pulmonary rehabilitation was also noted as a complex intervention, requiring equipment and specialist knowledge and skills to teach. Our co-designed intervention components focus on cognitive change by providing simple, high-level advice which is deliverable in the community-setting.

#### Promoting physical activity - Move more, sit down less

Our Steering Group highlighted that *anything* that people can do to remain active is beneficial. Allied Health Professionals noted that promoting exercise would be unhelpful due to associations with sport, but all Groups agreed that promoting breathless people to move more was key for improving resilience to breathlessness. Understandably, when breathlessness is uncontrolled, people experience this as an indicator of poor health. People may become breathless even doing very little physical activity (e.g. eating) and family members may discourage activities which make their loved one breathless. Stakeholders in all Groups endorsed messaging that becoming breathless is an expected response to exertion – and safe – and is important, in order to promote activity.

#### Pacing

Pacing, prioritisation of activities and planning are key strategies for promoting movement^39^. Pacing is intended to promote activity, but avoid ‘boom and bust’ cycles which see people over-exert when they feel well and do ‘too much.’ Promotion of physical activity was identified as problematic by the Doctors Group and Allied Health Group, because the normal thing to do in context of illness is to ‘rest.’ Family members may also encourage rest because it can be distressing to see the person get breathless. Suggestions that family members accompany the person with breathlessness in activities (e.g. short walk) reinforces feelings of safety.

### Living with breathlessness

Our Lived Experiences Group reported a range of methods which they used to promote calm, either for themselves or as a carer for a person with breathlessness. Massage, sipping a drink, listening to music and aromatherapy were all mentioned as helpful methods. These approaches are consistent with evidence indicating that distraction and relaxation techniques help breathing control^40^.

Our Lived Experiences Group also highlighted contexts for intervention delivery which they felt worsened breathlessness, including smoke (of any kind), air pollution and feeling crowded. Stakeholders in all Groups thought it would be helpful to include *Dos and Don’ts* explaining that common risk factors should be avoided if possible. Guidance on food and drink was also thought to be essential for delivering healthcare advice in context of health beliefs. Not including reference to food when providing health advice was thought to diminish credibility. Although no food group is known to worsen breathlessness, Doctor stakeholders report that their patients sometimes complain that dairy worsens their breathing. By contrast, there is emerging evidence that menthol may have a therapeutic effect^41^. Together with our stakeholders, we agreed that food advice should focus on gaining good nutrition. Because of reports from our Nurses and Allied Health Group that sometimes people avoid food, because the act of eating makes them breathless, we encourage good nutrition, regular small meals – and soft foods. Early evidence also indicates that menthol reduces dyspnoea on exertion in patients with chronic breathlessness, with minimal risk of adverse events. Use of menthol (e.g. sucking a mint) was therefore included as a recommendation^42^.

### Mode of delivery

Accredited Social Health Activists (ASHA) were identified by our realist review as a potential workforce for delivery of breathlessness education in communities. ASHA are lay health workers employed by the Government of India, who provide essential health services in communities across India, including health education and promotion. We convened an ASHA stakeholder group to hear their views about our breathlessness theory and about a potential role in delivery of breathlessness education. Our ASHA group confirmed that breathlessness is common in communities they serve and were supportive of the intervention. ASHA are members of the communities they serve and may share unhelpful beliefs regarding breathlessness. However, the Group confirmed that education regarding identifying the difference between breathing problems and 3D Breathlessness would be helpful. A *give it a go* approach was promoted by our Allied Health Group.

ASHA have a structured educational program. ASHA trainers are provided detailed educational materials including teaching sessions and deliver training to ASHA workers, along with core educational materials. Pain is currently the only symptom included in the ASHA Induction manual, the key text for ASHA education. Our ASHA group agreed that breathlessness education would be helpful as part of their basic training manual and that ASHA trainers would require additional information. The Project Group and Steering Group agreed that our educational intervention should directly target AHSA workers and educators, also providing them with resources that they can distribute in their communities.

### Co-design outputs

At the end of our project, we had developed breathlessness educational resources for ASHA workers, people with breathlessness and their families. Our prototype breathlessness resources are presented in Box 2 and are available here.

Box 2 Overview of Breathe-India co-designed breathlessness resources **1) A new language for breathlessness in India ‘3D Breathlessness - Daily, Distressing, Disabling, breathlessness’**

Drawing upon evidence generated, stakeholder and expert views, we present our working definition as; 3D Breathlessness - Daily, Distressing, Disabling breathlessness. This description aims to capture; the severity of the psychological impact of breathlessness (distressing), the physical consequences (disabling) and the pervasive presence of breathlessness in people’s lives (daily. The words used are non-technical, are intended to be meaningful to people experiencing breathlessness and translatable into other languages in India. Removal of reference to optimal management of disease acknowledges that cause(s) of breathlessness may not be diagnosed or treated and encourages breathlessness as a therapeutic target, in parallel with treatment of underlying pathology. The term 3D Breathlessness could replace terms like “Chronic Breathlessness Syndrome”, or “Persistent Breathlessness” due to its utility, impact and ready understanding,

**2) Summary of 3D Breathlessness and Acute Breathing Problems**

We prepared short one-page summaries of 3D Breathlessness and Acute Breathing Problems, highlighting differences and aimed at inclusion in ASHA basic training (Induction Module/Handbook). Identification of ‘red flags’ promote health seeking for medical treatment. Key information about 3D Breathlessness and self-management regards i) how to regain control (facial cooling, finding a comfortable position, rectangle breathing), ii) encouragement to Move More (breathlessness on exertion is normal/safe, movement helps gain or maintain strength and iii) avoid risk factors where possible (smoke, pollution) and encourages mask use. This resource has been agreed with ASHA trainers but needs testing with ASHA workers.

**3) Educational curriculum for ASHA trainers**

We prepared a short educational curriculum providing detailed information supporting delivery of education in relation to the 3D Breathlessness Summary. Teaching materials include educating ASHA trainers about potential barriers identified (e.g. cool air and association with poor health) and strategies to acknowledge and address concerns. A teaching curriculum is provided which teaches and tests ASHA knowledge of how to distinguish between Acute Breathing Problems in need of medical attention and 3D Breathlessness where self-management can also be taught. ASHA trainers helped us prepare the content, especially with language used. This content was deemed to be understandable and helpful, but will need translating into local languages. An English version is ready for testing in context as part of ASHA training to ASHA workers.

**4) Living with 3D Breathlessness (leaflet, video)**

Finally, we prepared a 3-part leaflet (with associated videos) aimed at people with breathlessness and family members. *Living with 3D Breathlessness* has three parts; 1) Regaining Control, 2) Moving More and 3) Living with 3D Breathlessness. We worked with illustrators in India and the United Kingdom to develop images which were appropriate to context and which reinforced educational messaging (e.g. an older woman out in the community using a walking aid, with family member in support). Each intervention component includes a ‘did you know?’ section which addresses common concerns. Three videos were also developed using the content of the leaflets, with narration.

## Discussion

Breathlessness is prevalent in communities in India, caused a range of medical and non-medical factors. It is prioritised by people with lived experiences, because it has widespread impacts on their wellbeing and health. It is also viewed as a priority by health workers, who themselves can feel distress at not being able to help breathless people. However, symptom-focussed interventions are not commonly available in health services in India, despite evidence of helpful interventions in HICs.

We worked with a range of stakeholders to co-design a breathlessness intervention of evidence-based components, for delivery in the community setting in India. Our interventions apply our programme theories and are focussed on cognitive-change and practical breathlessness self-management approaches which can be delivered at low-cost, within existing community healthcare infrastructure in India. Our interventions include components which are evidence-based and endorsed by the International Primary Care Respiratory Group. Our project has highlighted the complex health beliefs and behaviours which contextualise local understandings of breathlessness and which may influence intervention success in India. For example, we identified, cultural associations between cold and poor health which must be addressed when promoting facial cooling as a helpful breathlessness self-management approach. Similar work is now needed in additional contexts (e.g. Sub-Saharan Africa) where breathlessness interventions are underdeveloped and cultural health beliefs differ^43^.

Daily, Distressing, Disabling breathlessness (shortened to 3D Breathlessness) is an important new language for breathlessness in LMICs. Clinical definitions of breathlessness (e.g. chronic breathlessness syndrome^2^ all have the presumption that the underlying cause of breathlessness has been identified and optimally treated. Our work has demonstrated that breathlessness in India may have multiple causes, some or all of which may not have been diagnosed or treated. 3D Breathlessness is a definition which can be used to challenge health beliefs that disabling breathlessness is ‘normal’ and to promote appropriate health-seeking.

In 2024, the European Respiratory Society evaluated effectiveness evidence for breathlessness self-management and concluded that further clinical research was needed, with only tentative recommendations for implementation for some interventions (e.g. yoga) in the presence of a trained health worker^44^. By contrast, our findings support that a ‘give it a go approach’ for low-cost, low-harm interventions which stakeholders felt are likely to be beneficial, irrespective of grade of evidence. Breathlessness self-management interventions (e.g. handheld fan^18^ are commonly low-cost at the point of delivery, perceived as helpful by users and with few adverse events. The ‘know-do gap’ is the failure to translate what is known to work in patient care and is a big barrier to global health equity^45^. Our project adds to evidence that international collaborations and participatory methods are important mechanisms regarding how co-design can translate available evidence into developing acceptable, feasible, and adaptable health solutions in different settings.

Our Breathe-India interventions are evidence-based, but Implementation-effectiveness hybrid projects are needed to test our implementation strategies and ensure that effectiveness is not lost during implementation^46^. The initial step will involve translating the outputs into Kannada, the state language of Karnataka, followed by feasibility testing ahead of a hybrid Type 3 study. Although components of our intervention are similar to those used in HICs, given the nature of the problem in India, a breathlessness intervention may have much wider socioeconomic impacts in a low-resource country. Implementation-effectiveness approaches should explore whether the intervention can be delivered (acceptability, sustainability) and whether the intervention improves patient and family outcomes.

### Strengths and limitations

A key strength of our project was our participatory approach which allowed us to incorporate the views of international breathlessness experts with a wide range of stakeholders in India concerned with breathlessness. We were able to involve stakeholders with varied clinical experiences, delivery of community health interventions and lived experience, with men and women both well represented. Our realist approach also allowed us to incorporate a wide range of scientific and non-scientific data sources to explore the complex subject of breathlessness management in India. A limitation of our work is that we have not yet been able identify any changes which may yet be required to our interventions, when delivered in context, nor evaluated impact on patient and family outcomes. Finally, ASHA workers emerged as being important for delivery of breathlessness education. Although we were able to assemble an ASHA Stakeholder Group unexpectedly, it included only six people. It will be important to include more ASHA in future development and testing of our breathlessness resources.

## Conclusion

Breathlessness interventions developed in HICs may be used in India, but unhelpful beliefs must be addressed at the point of delivery. We co-designed the first multicomponent breathlessness resources aimed at implementing breathlessness education in a low-resource country. Implementation-effectiveness evaluation is now needed to test if it can be implemented and to evaluate patient and family outcomes.

## Supplementary information

Supplementary Information

## Data Availability

No datasets were generated or analysed during the current study.
