# Supplementary material for: Implementing breathlessness self-management in low- and middle-income countries: co-design of breathlessness self-management resources for use in India
Source: NPJ Prim Care Respir Med. 2025 Nov 24;35:55. doi: 10.1038/s41533-025-00458-2 (PMC12644879; doi:10.1038/s41533-025-00458-2)
Supplement: Supplementary file 1 [file 41533_2025_458_MOESM1_ESM.odt]

Stakeholder Group Composition

- Doctors Group (n=9), meetings; 3

Men: 4

Women: 5

Expertise: Palliative Care, Pulmonology, General Practice, Cardiology

Key roles: Evidence appraisal, (affirm and challenge), Theory development, Intervention development

Facilitator: Prof Salins

- Nurses, Allied Health Professionals Group (n=6), meetings; 3

Men: 0

Women: 6

Expertise: Physiotherapy, Palliative Nursing, Pulmonary rehabilitation

Key roles: Evidence appraisal, (affirm and challenge), Theory development, Intervention development

Facilitator: Dr Rao

- Lived Experiences Group (n=9), meetings; 3

Men: 3

Women: 6

Expertise: Lived experiences as patients and carers

Key roles: Evidence appraisal, (affirm and challenge), Theory development, Intervention development

Facilitator: Dr Bhat

- Accredited Social Health Activist (n=6), meetings; 2

Men: 0

Women: 7

Expertise: Community Health training and delivery

Key roles: Intervention development
